# Multiomic analysis revealed the regulatory role of the *KRT14* gene in eggshell quality

**DOI:** 10.3389/fgene.2022.927670

**Published:** 2022-09-22

**Authors:** Yan Wu, Yanyan Sun, Hao Zhang, Hongwei Xiao, Ailuan Pan, Jie Shen, Yuejin Pu, Zhenhua Liang, Jinping Du, Jinsong Pi

**Affiliations:** ^1^ Institute of Animal Husbandry and Veterinary, Hubei Academy of Agricultural Science, Wuhan, China; ^2^ Hubei Key Laboratory of Animal Embryo and Molecular Breeding, Wuhan, China; ^3^ Institute of Animal Sciences of CAAS, Beijing, China

**Keywords:** laying hen, eggshell, transcriptome, proteome, KRT14 gene

## Abstract

**Background:** Eggshell strength and thickness are critical factors in reducing the egg breaking rate and preventing economic losses. The calcite biomineralization process is very important for eggshell quality. Therefore, we employed transcriptional sequencing and proteomics to investigate the differences between the uteruses of laying hens with high- and low-breaking-strength shells.

**Results:** A total of 1,028 differentially expressed genes (DEGs) and 270 differentially expressed proteins (DEPs) were identified. The analysis results of GO terms and KEGG pathways showed that most of the DEGs and DEPs were enriched in vital pathways related to processes such as calcium metabolism, hormone and amino acid biosynthesis, and cell proliferation and apoptosis. Several DEGs and DEPs that were coexpressed at mRNA and protein levels were verified. *KRT14* (keratin-14) is a candidate gene (protein) obtained by multiple omics analysis due to the fold difference of *KRT14* being the largest. After the overexpression of *KRT14* in uterine epithelial cells, the expressions of *OC116* (ovocleididin-116), *CALB1* (calbindin 1), and *BST1* (ADP-ribosyl cyclase 2) were found to be increased significantly, while the expression of *OC17* (ovocleididin-17) was found to be decreased significantly.

**Conclusion:** In summary, this study confirms that during normal calcification, there are differences in ion transport between the uterus of hens producing high-breaking-strength eggshells and those producing low-breaking-strength eggshells, which may help elucidate the eggshell calcification process. The *KRT14* gene may promote calcium metabolism and deposition of calcium carbonate in eggshells.

## Background

The ability of chickens to produce large numbers of eggs over an extended laying cycle is very essential for the poultry industry. The eggshells of birds play an important role in determining the physical and antimicrobial defenses of eggs and regulating the exchange of metabolic gases and water (Hincke et al., 2012). Eggshell quality is very important for hatchability (Hrnčár et al., 2012), food safety, and egg marketing (Park and Sohn, 2018). Therefore, during the entire laying period, improving the sustainability of egg quality (especially eggshell strength) is very important for the poultry industry. Many factors, including health status, age, nutrition, and housing system, affect the productive performance and egg quality of laying hens (Vlčková et al., 2019). Eggs with low eggshell strength are easily damaged during collection, storage, and transportation. On average, broken eggs account for 7.5% of eggs produced, which can lead to significant economic losses (Roland, 1988). The eggshell structure is complex and has important biological functions for protection of the inner content of eggs after deposition, as well as for chicken embryo development (Hincke et al., 1999). The eggshell contains 95% calcium carbonate and 3.5% organic matrix proteins (Marie et al., 2014). The eggshell is formed in the uterus, which is a segment of the oviduct of laying hens. The process of eggshell formation can be divided into three crucial periods: initiation of crystal growth, linear crystal growth, and termination of mineralization (Marie et al., 2015). The eggshell is composed of a bilayered membrane, mammillary layer, palisade layer, vertical crystal layer, and cuticle layer (Solomon, 1991).

Previous studies have shown that there are some differences in the ultrastructure between high-strength eggshells and low-strength eggshells (Ahmed et al., 2005; Stefanello et al., 2014; Zhang et al., 2017). The ultrastructure of eggshells is related to matrix proteins which regulate the morphology, growth kinetics, and crystallographic orientation of calcite crystals (Nys et al., 2004; Nys et al., 2010); these proteins include ovocleidin-116, ovocleidin-17, ovocalyxin-32, and ovocalyxin-36 (Hincke et al., 1995; Hincke et al., 1999; Gautron et al., 2001; Gautron et al., 2007). Matrix proteins have been shown to participate in the biomineralization of the eggshell (Mann, 2015). However, most of these discoveries only provide genes expressed in certain segments of the chicken oviduct (which is the principal organ for egg development); the overall mechanisms of eggshell development have not been determined yet. Similar to other economically important traits, eggshell strengthening remains relevant during the whole period of productive life for laying hens and generally deteriorates with age (Sirri et al., 2018; Crosara et al., 2019). The decline in eggshell quality is still one of the main reasons for replacing commercial flocks (Bain et al., 2016). Thus, clarifying the genetic basis and molecular mechanisms underlying eggshell strengthening at different chicken breeds and laying stages is very important for breeders. Therefore, we studied the differences in the gene expression profiles of the uteri of laying hens producing high- and low-breaking-strength eggshells during peak laying periods in this study.

Keratin 14 (*KRT14*) is a predominant cytoplasmic intermediate filament protein, which belongs to the type I keratin family and forms a heterodimer with type II keratin *KRT5* (keratin 5). It is a prototypic marker of dividing basal keratinocytes where the *KRT14* gene is transcribed at high levels (Rose-Anne et al., 2007). Previous studies have shown that the mutation of *KRT14* and *KRT5* causes epidermolysis bullosa simplex (Bolling et al., 2011; Jankowski et al., 2014); *KRT14* may regulate the keratin turnover required for keratinocyte differentiation by interacting with receptor-interacting protein kinase 4 (*RIPK4*) (Smer et al., 2019). So far, the research on the *KRT14* gene in poultry has not been reported. *KRT14* is one of the genes with a large difference between the hard-eggshell group and the weak-eggshell group. In view of the important effect of *KRT14* in the process of keratinization, we preliminarily explored the role of *KRT14* in eggshell formation in this study.

## Methods

### Ethics statement

All animal experiments were conducted according to the guidelines established by the Regulations for the Administration of Affairs Concerning Experimental Animals (Ministry of Science and Technology, China, 2004). The tissues were collected from 43-week-old hens. The hens were raised under free food intake and were humanely killed in the laboratory. All experimental protocols were approved by the Institute of Animal Husbandry and Veterinary of Hubei Academy of Agricultural Science.

### Samples collected

Three eggs were collected from each of 1,820 hens from an experimental farm in Wuhan, China (Jianghan chicken, which is 43 weeks old and is a local chicken in China). Eggshell breaking strength, eggshell thickness, and egg weight were assessed. The eggshell thicknesses of eggs were tested using an electronic digital micrometer (Deli, Deli Group Co., Ltd, China). Eggshell breaking strength was assessed using an eggshell strength meter (NFN388, FHK Fujipin Co., Ltd, Japan). Eggs were weighed using an electronic balance (ES-E, Tianjin de ante Sensing Technology Co., Ltd, Tianjin, China).

The uteri of hens producing eggs with extreme eggshell thickness and eggshell breaking strength were collected immediately after oviposition for 6 h from six hens after being slaughtered (the hens were manually killed by the cervical dislocation method) in the laboratory. Three uterus samples were collected from the hard-eggshell group (HE group or thick group, exhibiting eggshells were strong and thick) and three uterus samples from the weak-eggshell group (LE group or thin group, exhibiting eggshells were weak and thin). The egg parameters of the six hens are listed in Table 1. Then, the uteri were frozen in liquid nitrogen for RNA-seq analysis and experimental validation.

**TABLE 1:** Egg parameters for the sequenced hens.

Sample	Eggshell strength (kg/cm2)	Egg thickness (mm)
HE 1	5.16	0.36
HE 2	5.41	0.38
HE 3	5.15	0.36
LE 1	1.54	0.13
LE 2	1.54	0.11
LE 3	1.32	0.11

### Transcriptome sequencing

Total RNA was isolated with TRIzol reagent (Invitrogen, United States). After total RNA was isolated from each individual sample and assessed, the concentration, quality, and integrity were determined by a NanoDrop Spectrophotometer (NanoDrop Technologies, Wilmington, DE, United States). A total of 5 μg RNA from each sample was employed for sequencing and library construction, which were performed at Novogene Biotech Co., Ltd. (Beijing, China).

First-strand cDNA was synthesized using random hexamer primer and M-MuLV Reverse Transcriptase, and then the RNaseH was used to degrade the RNA. Second-strand cDNA synthesis was subsequently performed using DNA Polymerase I and dNTP. The remaining overhangs were converted into blunt ends via exonuclease/polymerase activities. After adenylation of 3’ ends of DNA fragments, adapters with hairpin loop structure were ligated to prepare for hybridization. In order to select cDNA fragments of preferentially 370–420 bp in length, the library fragments were purified with the AMPure XP system (Beckman Coulter, Beverly, United States). Then, after PCR amplification, the PCR product was purified by AMPure XP beads, and the library was finally obtained. After the construction of the library, the library was initially quantified by Qubit2.0 Fluorometer, then diluted to 1.5ng/ul, and the insert size of the library was detected by Agilent 2,100 bioanalyzer (Agilent Technologies, Palo Alto, CA, United States), and Also, being sequenced using the Illumina PE150 platform (Illumina, San Diego, CA) with paired-end 150 bp reads. Sequences were mapped to the chicken reference genome database from the NCBI database (ftp.ncbi.nlm.nih.gov/genomes/genbank/vertebrate_other/Gallus_gallus/latest_assembly_versions/GCA_000002315.5_GRCg6a/) with TopHat2 (Dou et al., 2016).

Differential expression analysis of two groups (three biological replicates per condition) was performed using the DESeq2 R package (Version 1.20.0). DESeq2 provides statistical routines for determining differential expression in digital gene expression data using a model based on the negative binomial distribution. *p*-value <0.05 and |log2 (foldchange)| >0 were set as the thresholds for significantly differential expression.

Gene Ontology (GO) enrichment analysis of differentially expressed genes was implemented by the clusterProfiler R package (version 3.8.1), in which gene length bias was corrected. GO terms with corrected *p-value* less than 0.05 were considered significantly enriched by differentially expressed genes. KEGG is a database resource for understanding high-level functions and utilities of the biological system such as the cell, the organism, and the ecosystem, from molecular-level information, especially large-scale molecular datasets generated by genome sequencing and other high-throughput experimental technologies (http://www.genome.jp/kegg/). We used the clusterProfiler R package (version 3.8.1) to test the statistical enrichment of differential expression genes in KEGG pathways.

All RNA-seq data were uploaded to the NCBI Sequence Read Archive (https://www.ncbi.nlm.nih.gov/sra/PRJNA660090; SRA accession number: PRJNA660090).

### Protein extraction, TMT labeling, and LC-MS/MS analysis

Total proteins (the sample is the same as the RNA-seq) were extracted by the cold acetone method. The sample was ground individually in liquid nitrogen and lysed with lysis buffer (which contained 100 mM NH_4_HCO_3_ (pH 8), 8 M urea, and 0.2% SDS) and then ultrasonicated on ice for 5 min. After centrifuging at 12,000 g for 15 min at 4°C, the supernatant was transferred to a clean tube. Extracts from each sample were reduced with 10 mM DTT for 1 h at 56°C and subsequently alkylated with sufficient iodoacetamide for 1 h at room temperature in the dark. Then, samples were completely mixed with four times the volume of precooled acetone by vortexing and incubated at 20°C for at least 2 h. After being centrifuged, the precipitate was collected. After washing twice with cold acetone, the pellet was dissolved by dissolution buffer, which contained 0.1 M triethylammonium bicarbonate (TEAB, pH 8.5) and 6 M Urea.

A total of 120 μg of each protein sample was taken. The volume was made up to 100 μL with dissolution buffer. 1.5 μg trypsin and 500 μL of 100 mM TEAB buffer were added to each sample. Then, the sample was mixed and digested at 37°C for 4 h. After that, 1.5 μg trypsin and CaCl_2_ were added to each sample, and they were digested overnight. Formic acid was mixed with the digested sample, adjusted pH under 3, and then centrifuged at 12,000 g for 5 min at room temperature. The supernatant was slowly loaded to the C18 desalting column and washed three times with washing buffer (0.1% formic acid; 3% acetonitrile) and then eluted with some elution buffer (0.1% formic acid and 70% acetonitrile). The eluents of each sample were collected and lyophilized. 100 μL of 0.1 M TEAB buffer was added to reconstitute, and 41 μL of the acetonitrile-dissolved TMT labeling reagent was added, and then the sample was mixed with shaking for 2 h at room temperature. Then, the reaction was stopped by adding 8% ammonia. All labeling samples were mixed with equal volume, desalted, and lyophilized.

Mobile phase A (2% acetonitrile, adjusted pH to 10.0 using ammonium hydroxide) and B (98% acetonitrile) were used to develop a gradient elution. The lyophilized powder was dissolved in solution A and centrifuged at 12,000 g for 10 min at room temperature. The sample was fractionated using a C18 column (Waters BEH C18 4.6 × 250 mm, 5 μm) on a Rigol L3000 HPLC operating at 1 ml/min, and the column oven was set to 50°C. The elution gradient is listed in Table 2. The eluates were monitored at UV 214 nm, collected for a tube per minute, and combined into 10 fractions at last. All fractions were dried under vacuum and then reconstituted in 0.1% (v/v) formic acid (FA) in water.

**TABLE 2:** Proteins/genes differentially expressed in both the proteome and transcriptome.

Gene_id	Tran_id	Prot_id	GO_fun	kegg
CDH17	420225	A0A1D5NVS2	Cell adhesion	-
KRT14	408039	A0A1D5PZ89	Structural molecule activity	-
ANXA2	396297	P17785	Molecular function regulator	-
DKK3	396023	F1NRD7	Extracellular region	-
BET1	420563	E1BRC4	Binding	SNARE interactions in vesicular transport (map04130)
HTATIP2	424363	A0A1L1RLC7	-	-
LOX	396474	Q05063	Single-organism process	-
STAT1	424044	Q5ZJK3	Single-organism process	AGE-RAGE signaling pathway in diabetic complications (map04933)
PDLIM7	416362	A0A1D5PJC9	Binding	-
BPGM	418172	Q5ZHV4	Single-organism process	Metabolic pathways (map01100)
TFF2	418534	E1BZ37	-	-
CD109	421862	F1NX21	Extracellular region	-
P2RX7	771952	E1C6P3	Transporter activity	Calcium signaling pathway (map04020)
RRM2B	420253	A0A1D5PI47	Single-organism process	p53 signaling pathway (map04115)
FBLN1	373979	F1NX60	Extracellular region	-
ABCC3	422099	F1NM51	Membrane	ABC transporters (map02010)
SCIN	420588	A0A1I7Q413	Single-organism process	-
COMP	420120	E1C8N1	Extracellular region	Focal adhesion (map04510)
NT5DC3	427914	A0A1D5PNR2	-	-
MAP2	424001	A0A3Q2U4G6	Single-organism process	-

An EASY-nLCTM 1200 UHPLC system (Thermo Fisher) coupled with an Orbitrap Q Exactive HF-X mass spectrometer (Thermo Fisher) operated in the data-dependent acquisition (DDA) mode was used to perform Shotgun proteomics analysis, which was performed at Novogene Biotech Co., Ltd. (Beijing, China).

Proteins with fold change in a comparison ≥ 1.2 or ≤ 0.83 and unadjusted significance level *p* < 0.05 were considered to be differentially expressed. Gene Ontology (GO) and InterPro (IPR) analyses were conducted using the InterProscan-5 program against the nonredundant protein database (including Pfam, PRINTS, ProDom, SMART, ProSiteProfiles, and PANTHER) (Jones et al., 2014). The protein family and pathway were analyzed by the KEGG database (Kyoto Encyclopedia of Genes and Genomes). The STRING-db server (http://string-db.org/) based on the related species was used to predict the probable interacting partners. The probable protein–protein interactions were predicted using the STRING-db server (Franceschini et al., 2013) (http://string.embl.de/). The enrichment analysis of GO and KEGG was performed by the enrichment pipeline (Huang et al., 2009).

### Correlation analysis between proteomic and transcriptomic results

The differentially expressed genes (DEGs) and the differentially expressed proteins (DEPs) were identified separately, and Venn diagrams were plotted according to the counted results. Correlation analysis was performed by R (version 3.5.1), and the maps were drawn based on changes in the transcriptome and proteome analysis.

### Plasmid construction

Chicken *KRT14* (NM_001001311) was amplified and then digested with *KpnI* and *XhoI* (NEB, Ipswich, MA, United States) and cloned into the *pcDNA3.1 (+)* vector (Promega).

### Uterine epithelial cell isolation, culture, and transfection

The blood vessels, connective tissue, and fascia on the surface of the uterus were removed. The endometrial epithelial cells were gently scraped with a surgical blade. The scraped cells were collected and subjected to enzymatic digestion using collagenase type I with a final concentration of 1 mg/ml. The cells were filtered through a 74-μM mesh screen after enzymatic digestion for 60 min at 37°C and then washed and resuspended in culture media (DMEM/F12, 10% fetal bovine serum, and 1% penicillin and streptomycin). At last, the cell number was detected by the Trypan Blue method. The cells were seeded in 6-well culture plates with 1 × 10^6^ cells/well. The cells were cultured at 37°C with 5% CO_2_. The transfection assays were conducted when cells reached 80–90% confluence. The overexpression vector of *KRT14* and the *pcDNA3.1 (+)* vector were transfected into the cells by Lipofectamine 3,000 transfection reagent (Invitrogen, Carlsbad, California, United States). For each construct, experiments were carried out in triplicate.

### Quantitative real-time PCR analysis of differentially expressed genes

RNA samples from the three chickens used for the RNA-seq experiment were analyzed by Q-PCR. The concentrations of RNA samples were measured using a NanoQuant Plate (TECAN, Infinite M200PRO). Total cDNA was synthesized using a PrimeScript RT Reagent Kit with gDNA Eraser (Perfect Real Time) (TaKaRa, Dalian, China). qRT-PCR was performed with a LightCycle^®^ 480 II (Roche) in a final volume of 20 μL using THUNDERBIRD SYBR Q-PCR Mix (TOYOBO, Osaka, Japan). Each 20-μL reaction volume contained 10 μL of THUNDERBIRD SYBR Q-PCR Mix, 0.3 μL of each divergent primer, 1 μL of cDNA, and 8.4 μL of double-distilled H_2_O (ddH_2_O). The cycling conditions included an initial single cycle (98°C for 10 s) followed by 40 cycles (94°C for 15 s and 60°C for 30 s). β-actin was used as an internal control for expression of mRNA. The relative expression level was normalized and calculated using the 2^−ΔΔCt^ method (Livak and Schmittgen, 2001). All primers were designed using Primer 5 (listed in Supplementary Table S1).

### Western blot analysis

The uterine protein lysates were generated by RIPA lysis buffer (Beyotime, Beijing, China). The SDS-PAGE was used to extract and separate uterine proteins. Then, the uterine protein was transferred to PVDF membranes which were blocked with skim milk. After that, the antibodies for β-actin (1:1,000, Abcam, Cambridge, MA, United States) and differential proteins (1:2000–1:1,000, Abcam, Cambridge, MA, United States) were used for immunoblotting. The protein expression levels were compared to β-actin expression using ImageJ (Wayne Rasband, National Institutes of Health, United States; Version 1.42q).

### Statistical analysis

All data analyses was performed by GraphPad Prism 5 (San Diego, CA; Version 5). The experimental values are presented as the means ± SDs. The means of the groups were compared by Student’s t-test. *p-values* < 0.05 were considered to be statistically significant.

## Results

### Analysis of the DEGs in the uterus for HE and LE hens

**FIGURE 1:**
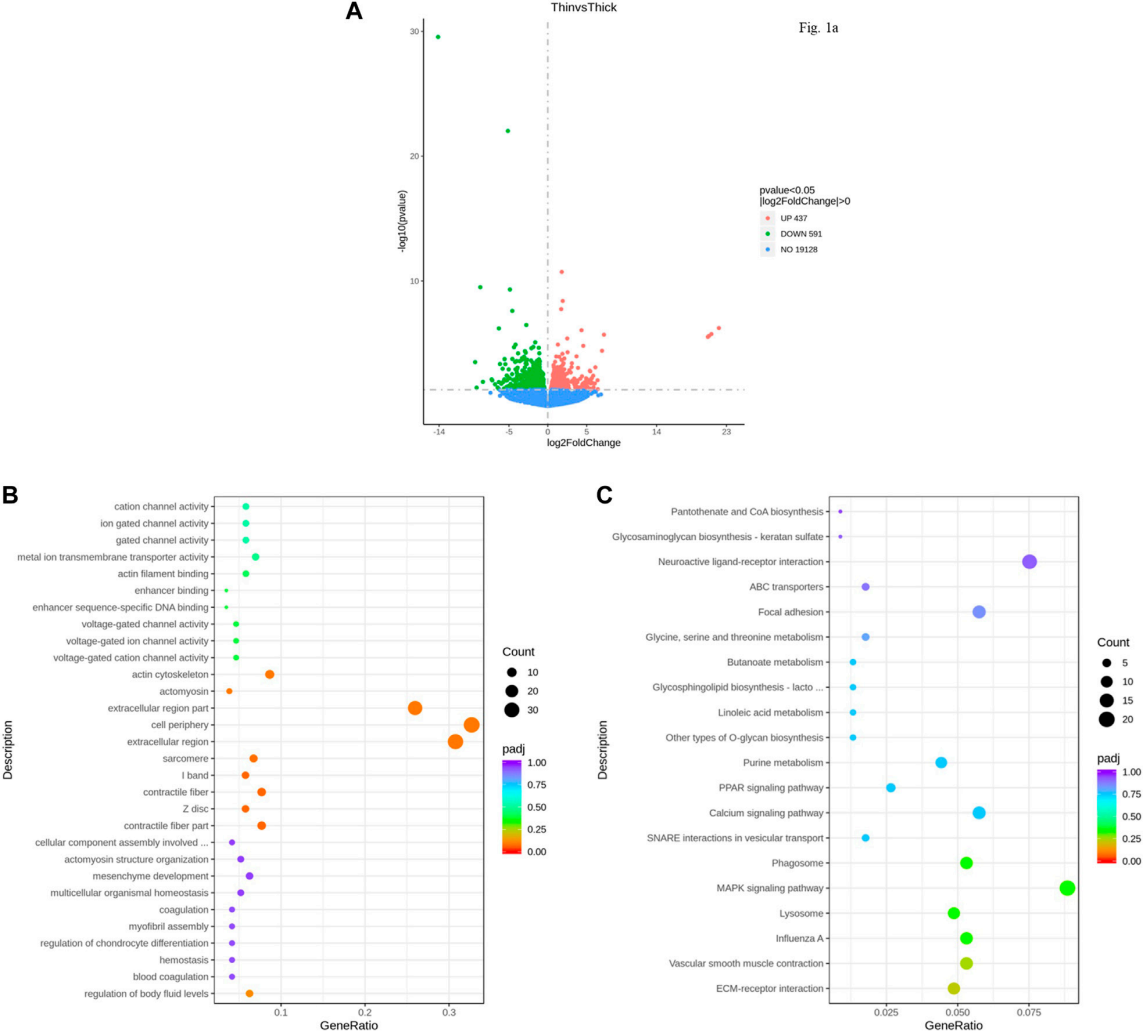
Differentially expressed genes (DEGs) identified for the uterus between HE and LE hens. **(A)**. Volcano plot of DEGs between HE and LE hens; **(B)**. GO analysis result of DEGs between HE and LE hens; **(C)**. KEGG analysis result of DEGs between HE and LE hens.

A total of 353 million raw reads were obtained from the RNA-seq data. After the adapter and low-quality sequences were removed, clean data were obtained. The total number of reads generated from the six samples is shown in Supplementary Table S2. Each sample yielded more than 45 million raw reads. A total of 19,128 expressed genes were detected between HE and LE groups. DEGs were identified by log2 (foldchange) >0 and *p-values* < 0.05 (Supplementary Table S3). There were 1,028 annotated genes that were significantly different (*p < 0.05*) in the uterus between hard and weak groups (Supplementary Table S3). Of these genes, 437 genes were upregulated, and 591 genes were downregulated (LE vs. HE) (Figure 1A).

GO analysis of the DEGs of the six uterus samples indicated that they were enriched in cellular component, biological process, and molecular function for terms such as cell periphery, extracellular region, and actin cytoskeleton (Figure 1B). The top 20 results from KEGG analysis showed that some vital pathways related to calcium metabolism, cell proliferation, and apoptosis could also be influenced by the pathway in which these DEGs in the uterus were enriched, including the calcium signaling pathway, MAPK signaling pathway, and PPAR signaling pathway (Figure 1C).

### Protein identifications and DEP analysis by TMT proteome

To elucidate the mechanism underlying differences in eggshell strength, the differentially expressed proteins of the HE and LE groups were detected by TMT proteomics. Totally, 6,786 proteins were identified. There were 270 differentially expressed proteins between the LE and HE groups that were significantly different (Supplementary Table S4), including 161 upregulated proteins and 109 downregulated proteins (LE vs. HE).

**FIGURE 2:**
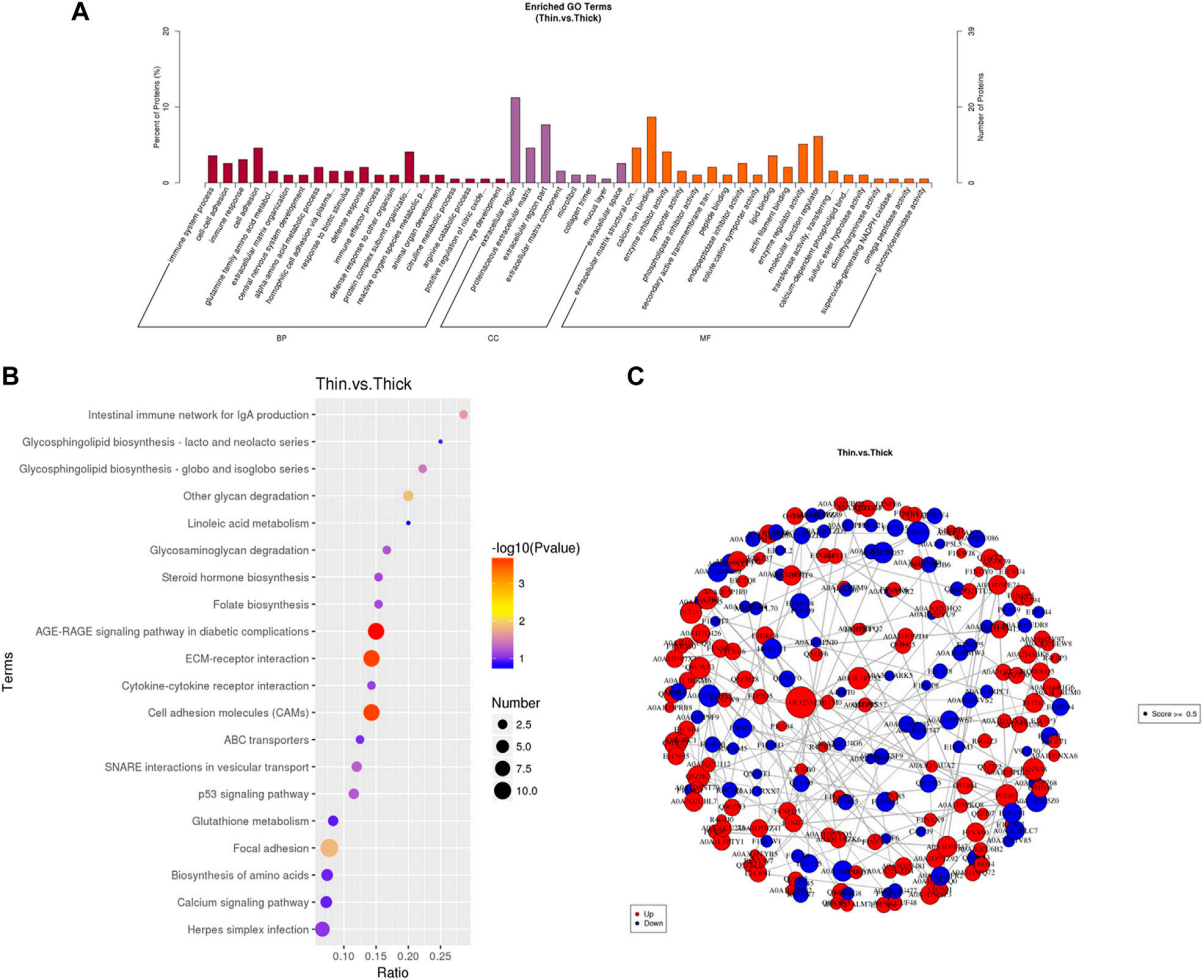
Differentially expressed proteins (DEGs) identified for the uterus between HE and LE hens. **(A)** GO analysis result of DEPs between HE and LE hens; **(B)** top 20 results of KEGG analysis of DEPs between HE and LE hens; **(C)** protein–protein interaction network of the DEPs.

GO analysis showed that the DEPs were primarily enriched in biological processes, molecular functions, and cellular components, including calcium ion binding, molecular function regulators, and extracellular regions (Figure 2A). The top 20 pathways of KEGG enrichment analysis further indicated that these DEPs were most enriched in some vital pathways related to processes such as calcium metabolism, hormone and amino acid biosynthesis, and cell proliferation and apoptosis; these proteins were involved in the calcium signaling pathway, cell adhesion molecules (CAMs), ECM–receptor interactions, the p53 signaling pathway, biosynthesis of amino acids, and steroid hormone biosynthesis (Figure 2B).

The protein–protein interaction (PPI) relationship was also revealed by the STRING database. To further prove the function of DEPs, we constructed a protein–protein interaction network of the DEPs (Figure 2C). Most of those genes were associated with each other.

### Combined analysis between differentially expressed transcriptomes and proteomes

**FIGURE 3:**
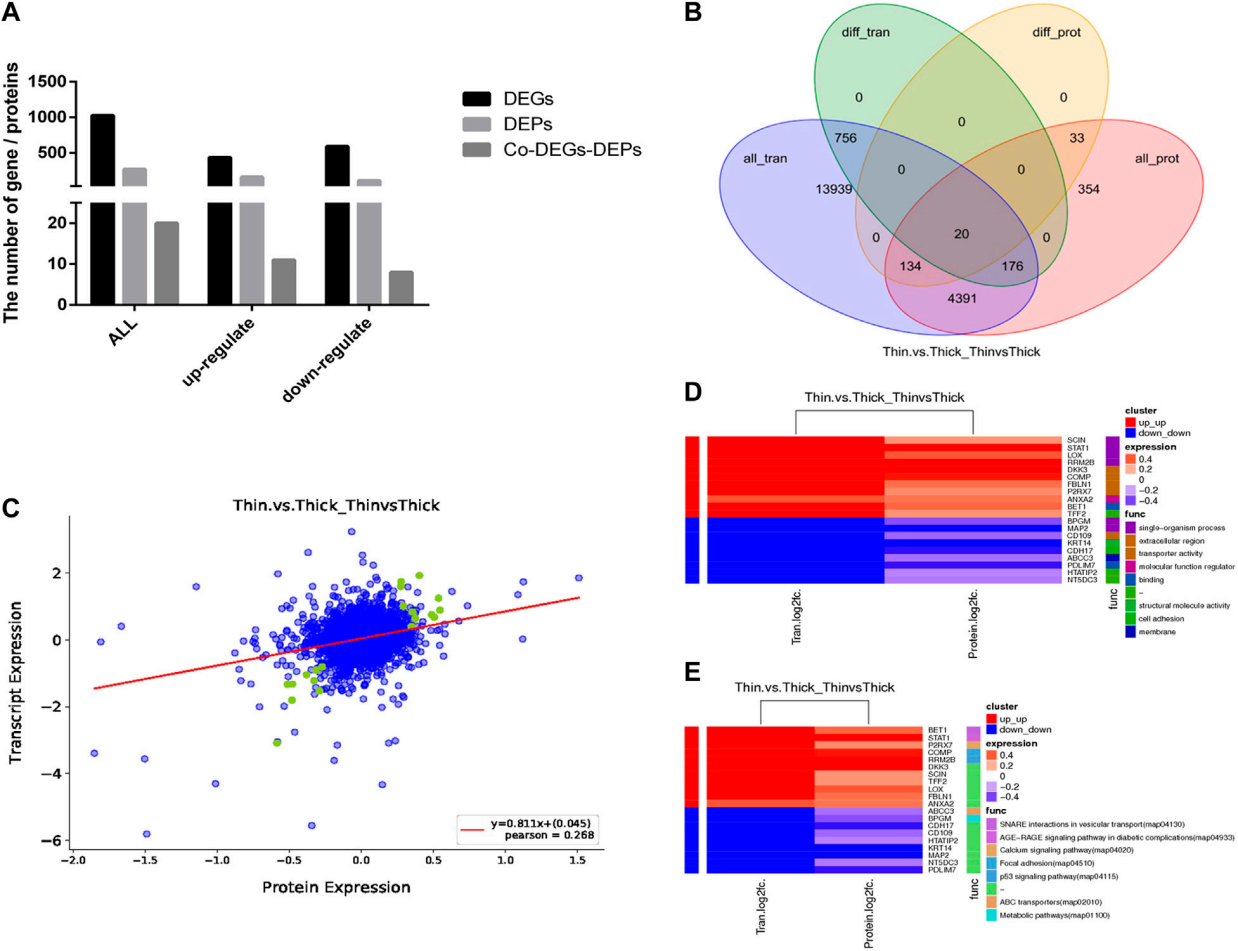
Integration and analysis of transcriptome and proteome between HE and LE groups. **(A)**. DEGs, DEPs, and Co-DEGs-DEPs between HE and LE groups; **(B)**. Venn diagram for DEGs and DEPs between HE and LE groups; **(C)**. Corr_plot of RNA-seq and TMT between HE and LE groups; **(D)**. Share GO analysis of RNA-seq and TMT between HE and LE groups; **(E)**. Share KEGG analysis of RNA-seq and TMT between HE and LE groups.

To elucidate the relationship between the transcriptome and proteome in the HE and LE groups, the two different omics datasets were integrated and analyzed. The results indicated that the number of DEGs and DEPs varied considerably in different eggshell-strength groups (Figures 3A–C), and some genes (or proteins) were differentially expressed at the gene level but not at the protein level, possibly because they were late-response genes. There were 20 genes and proteins detected at both the gene and protein levels (Table 2), and the regulation trend in mRNA and protein levels was matched. The GO enrichment analysis results showed that the correlated-expression DEGs/DEPs were primarily enriched in processes such as transporter activity, molecular function regulation, single-organism processes, membrane, and structural molecule activity (Figure 3D); meanwhile, KEGG enrichment analysis results showed that the correlated-expression DEGs/DEPs were primarily enriched in such processes as metabolic pathways, the p53 signaling pathway, focal adhesion, the calcium signaling pathway, and SNARE interactions in vesicular transport (Figure 3E).

### The validation of differentially expressed genes and proteins

**FIGURE 4:**
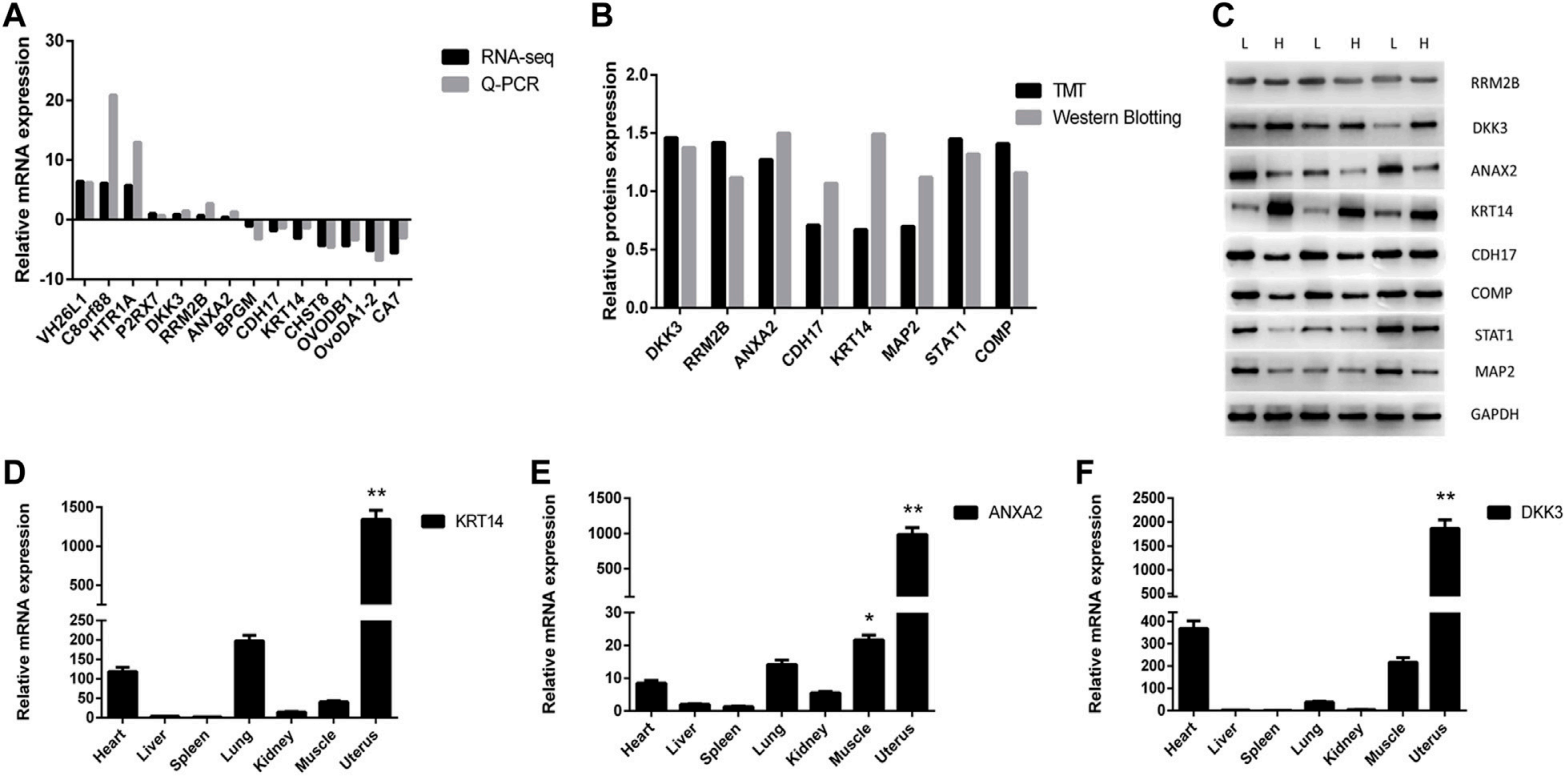
Identification and verification of differentially expressed gene and protein. **(A)**. Changes in DEG expression validated by qRT-PCR. Comparison results of the 14 mRNAs using the qRT-PCR and RNA-seq; **(B)** changes of DEP expression validated by Western Blot. Comparison results of the eight proteins using the Western Blotting and TMT; **(C)**. Western Blot result of part DEPs; **(D)** relative expression of *KRT14* in different tissues; **(E)** relative expression of *ANXA2* in different tissues; **(F)** relative expression of *DKK3* in different tissues.

The differentially expressed genes (DEGs) and differentially expressed proteins (DEPs) between LE and HE hens were identified. Seven upregulated and seven downregulated genes were randomly selected for verification via Q-PCR and Western blot, and eight proteins that were co-expressed at the mRNA and protein levels were randomly selected for verification by Western blotting. The results are shown in Figure 4A,B and supplement Figure 1. The log2 fold changes (LE/HE) were calculated based on the RNA-seq and Q-PCR results for DEGs and the TMT and Western blotting results for DEPs. The expression trends indicated that the two methods produced consistent results. Additionally, we investigated the relative expression levels of *KRT14, ANXA2*, and *DKK3* in different chicken tissues (i.e., heart, liver, spleen, lung, kidney, muscle, and uterus) (Figures 4C–E). In particular, the *KRT14*, *ANXA2*, and *DKK3* genes exhibited higher expression levels in the uterus than in other tissues, and these genes were expressed at the lowest levels in the spleens and livers of hens. Thus, the tissue expression profile of the *KRT14*, *ANXA2*, and *DKK3* genes demonstrated tissue specificity.

### Effects of KRT14 on the gene expression of eggshell matrix proteins, calcium transport, and calcium metabolism-related proteins

*KRT14* is one of the genes with a large difference between the hard-eggshell group and the weak-eggshell group. It was differently expressed both in transcription and protein level. Also, the fold difference of *KRT14* was the largest. So, *KRT14* was selected for further study.

**FIGURE 5:**
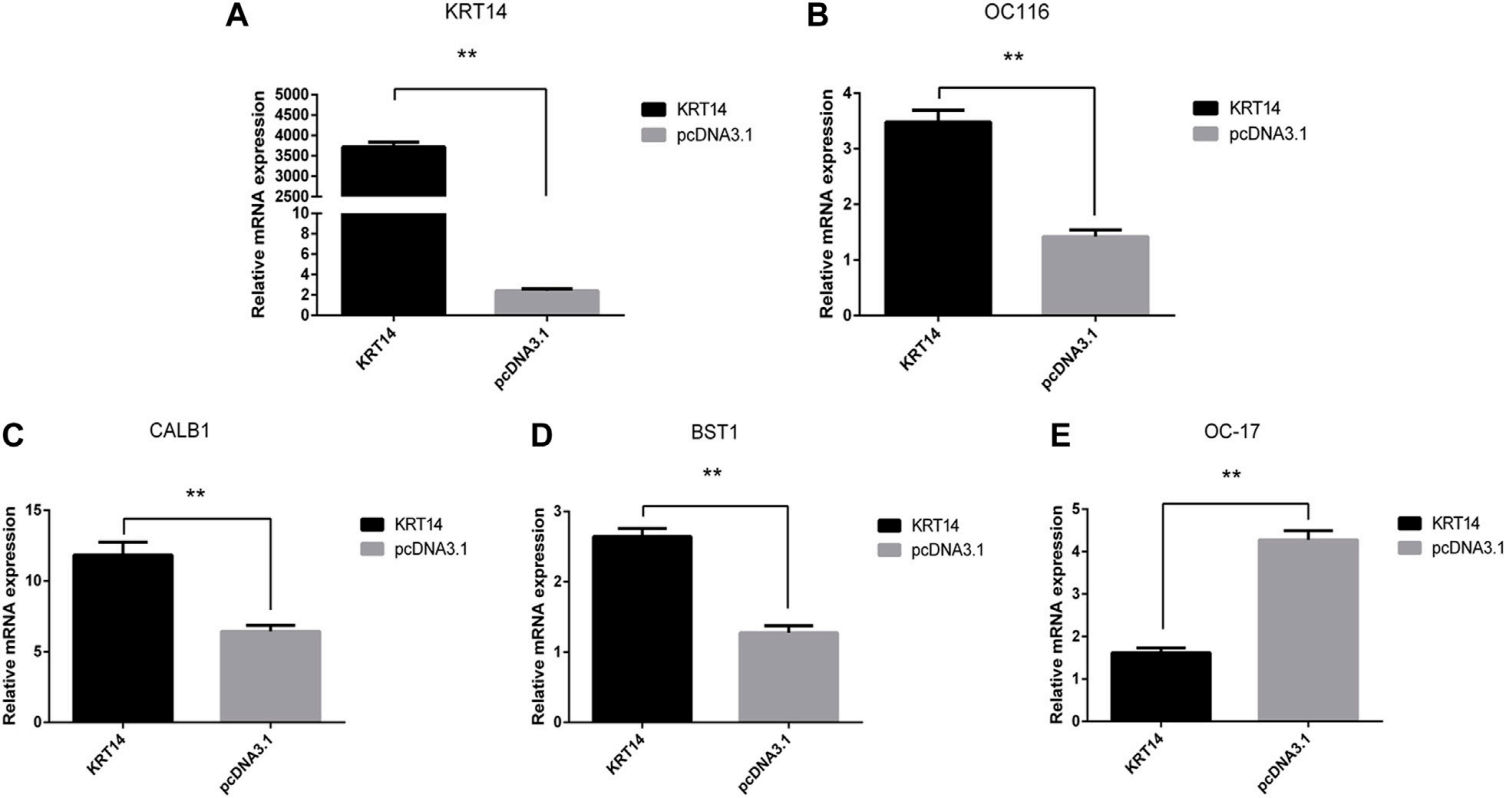
Effect of *KRT14* on calcium metabolism and deposition of calcium carbonate in eggshell. **(A–E)**: Expression levels of *KRT14, OC116, CALB1, BST1,* and *OC17* mRNA.

To verify the effect of the *KRT14* gene on eggshell quality, the gene expressions of eggshell matrix proteins, calcium transport, and calcium metabolism-related proteins in uterine epithelial cells were detected by Q-PCR. The results showed that the *KRT14* expression levels in the uterine epithelial cells were significantly increased after transfection with *KRT14-pcDNA3.1* (*KRT14*) (Figures 5A–E). After the overexpression of the *KRT14* gene, the mRNA expressions of *KRT14*, ovocleididin-116 (*OC116*) (the matrix protein), calbindin 1 (*CALB1*) (the calcium transport protein), and ADP-ribosyl cyclase 2 (also known as *BST1*, *CD157*) (calcium metabolism-related protein) were significantly increased (*p* < 0.01); the mRNA expressions of ovocleididin-17 (*OC17*) were significantly decreased (*p* < 0.01).

## Discussion

Eggshell formation can be divided into several stages (Li et al., 2018). The mammillary knob layer is the first calcified layer, which is formatted with the formation of the outer surface of the outer shell membrane, and its tips are embedded in the outer shell membrane (Carnarius et al., 1996). The palisade layer formed with the mammillary knobs fuse. The vertical crystalline layer consists of short crystals running perpendicular to the shell membrane which is the last calcified layer to be deposited (Solomon, 1991; Hunton, 2005). Previous research studies have shown that shell quality is dependent on the mammillary layer (Bain, 1992), and column organization of the palisade layer is one of the major determinants of rigidity of the shell, strength, and shell resistance of the eggs (Fathi et al., 2007). Large amounts of calcium ions and matrix proteins are required when eggshells are calcified in the uterus. During the calcification periods (including initiation, growth, and termination), calcium metabolism and uterine proteins exhibit changes. The quality of eggshell is determined by its ultrastructure (Rodriguez-Navarro et al., 2002), especially the palisade layer (Meyer et al., 1973). Therefore, previous studies mainly focused on the growth period when the palisade layer is formed (Jonchère et al., 2010; Brionne et al., 2014).

It is reported that the initiation stage of calcification determines the strength of the eggshell (Zhang et al., 2019). The slit guidance ligand 3 (*SLIT3*) was validated as a candidate gene for eggshell strength (Gao et al., 2022). However, during synchronous calcification periods, few differences in the uterine between hens with high eggshell breaking strength and those with low eggshell breaking strength have been reported, and the genetic regulation of calcification and eggshell strength are still poorly understood. In this study, the differentially expressed gene and differentially expressed protein were identified in the uteri of LE and HE hens. The results showed that the numbers of DEPs were significantly lower than those of DEGs. Fewer proteins were detected than genes in the Venn diagram analysis, probably due to modification and activation at the protein level, the short length of the sample treatment period or the limitations of protein detection technologies (Ma et al., 2020).

The KEGG had been used to classify DEGs and DEPs. The results showed that most DEGs and DEPs were enriched in vital pathways related to calcium metabolism, hormone, amino acid biosynthesis, cell proliferation, or apoptosis. Thus, we hypothesized that quality of eggshells is closely related to deposition of calcium ions. The GO analysis showed that many DEGs and DEPs were enriched in ion transport functions or cytoskeletal functions associated with eggshell calcification. These results implied that there were differences in ion transport between the uteri of hens producing high- or low-breaking-strength eggshells during normal calcification, which helped clarify the eggshell calcification process. In addition to the common ion transport genes found in previous reports, some novel genes were also described in this study (e.g., *ANXA2, DKK3,* and *KRT14*).

Annexin A2 (*ANXA2*) is a multifunctional calcium (Ca) and phospholipid-binding protein that is expressed in a wide spectrum of cells, including those participating in the inflammatory response (Dallacasagrande and Hajjar, 2020). Dickkopf 3 (*DKK3*) is a secreted protein that belongs to the Dkk family, and it is encoded by the orthologous gene *REIC*. It was reported that *DKK3* is a physiological ER stressor in the mouse adrenal gland (Fujita et al., 2020). Keratin-14 (*KRT14*) is a key regulator of spheroid integrity, mesothelial attachment, and invasion into the submesothelial matrix. Keratin-14 (*KRT14*)-positive leader cells mediate mesothelial clearance and invasion by ovarian cancer cells (Bilandzic et al., 2019). In this study, *ANXA2*, *KRT14*, and *DKK3* were determined to be significantly upregulated in LE hens compared to HE hens. In addition, the mRNA expression levels of the *ANXA2*, *KRT14*, and *DKK3* genes were significantly elevated in the uterus. So, it was presumed that *ANXA2*, *KRT14*, and *DKK3* may be related to the quality of eggshells.

Eggshell biomineralization is an extremely complex biological process, which is regulated by multiple genes and signal pathways (Sah et al., 2018; Khan et al., 2019). Ovocleididin-17 is the most vital protein regulating the deposition of calcium carbonate in eggshells. It plays an important role in the formation of eggshells and regulates the formation process of calcite crystals in the eggshell matrix (Maxwell and Hincke, 2013). Ovocleididin-116 is a major protein in chicken eggshell matrices (Mann et al., 2006), which is localized throughout the palisade layer and the mammillary layer (Hincke et al., 1999). *In vitro* crystallization experiments indicated a direct interaction of *OC116* with calcium carbonate (Hernández-Hernández et al., 2008). Calbindin 1 (*CALB1*) is the carrier of calcium ions in uterine epithelial cells (Sun et al., 2016). During the calcification process, the expression of the *CALB1* gene in the uterus was increased significantly in order to meet the mineralized calcium requirement of the eggshell (Jonchère et al., 2012). ADP-ribosyl cyclase 2, also known as *BST1* or *CD157*, could produce cyclic ADP‐ribose involved in regulating calcium homeostasis and plays an important regulatory role in calcium metabolism (Lee, 2001; Yamamoto‐Katayama et al., 2002). In this study, after the overexpression of the *KRT14* gene, the expression of *OC116*, *CALB1*, and *BST1* was significantly increased, while the expression of *OC17* was significantly decreased. The abovementioned results suggested that *KRT14* may promote calcium metabolism and deposition of calcium carbonate in eggshells.

## Conclusion

Based on the transcriptome and proteome analysis, our results were consistent with those of previous studies, which indicated that the initiation period of calcification determines the eggshell strength in the uterus. During normal calcification, there are differences in ion transport between the uterus of hens producing high-breaking-strength eggshells and those producing low-breaking-strength eggshells, which may help elucidate the eggshell calcification process. *KRT14* may promote calcium metabolism and the deposition of calcium carbonate in eggshells.

## Data Availability

The data that support the findings of this study are openly available in NCBI Sequence Read Archive at https://www.ncbi.nlm.nih.gov/bioproject/?term=PRJNA660090, SRA reference number PRJNA660090. The mass spectrometry proteomics data have been deposited to the ProteomeXchange Consortium (http://proteomecentral.proteomexchange.org) via the iProX partner repository with the dataset identifier PXD021449.

## Abbreviations

HE, hard-eggshell; LE, week-eggshell; DEGs, differentially expressed genes; DEPs, differentially expressed proteins; ANXA2, annexin A2; DKK3, Dickkopf 3; KRT14, keratin-14
