# Supplementary material for: Multiomic analysis revealed the regulatory role of the KRT14 gene in eggshell quality
Source: Front Genet. 2022 Sep 22;13:927670. doi: 10.3389/fgene.2022.927670 (PMC9536113; doi:10.3389/fgene.2022.927670)
Supplement: Supplementary file 2 [file Table1.DOCX]

**Supplementary Table S1: Primer sequences**

| Name | Sequence (5'-3') |
| --- | --- |
| VH26L1-F | 5’- GCCCACTCGTCTCCTCCC-3’ |
| VH26L1-R | 5’-GCTCTGCCCGTTGTCCCT-3’ |
| C8orf88-F | 5’- CTCCAACCAGCACTACCT-3’ |
| C8orf88-R | 5’-CTTTCTTTTTTCGCATCC-3’ |
| HTR1A-F | 5’- AACAACACTACCTCCCCA-3 |
| HTR1A-R | 5’-CTGCCCCAGAGTCCACTT -3’ |
| P2RX7-F | 5’- GATGACAAAACAAGAACCC -3’ |
| P2RX7-R | 5’-CACAGCCAAACCAAAATAG -3’ |
| DKK3-F | 5’- AACTTACCTCCCACCTACC -3’ |
| DKK3-R | 5’- ACAGCATTCAACATCTCGT -3’ |
| RRM2B-F | 5’- AGGGCGAAGGGGAGAGCAC -3’ |
| RRM2B-R | 5’- ATGACGAACCGACGGGGAT -3’ |
| ANXA2-F | 5’- TCTGCTCTGTCAGGTCAT-3’ |
| ANXA2-R | 5’- TTCCAGTTCTGTTTTGTA-3’ |
| BPGM-F | 5’- GAAGCTCAAAACTGTGGC-3’ |
| BPGM-R | 5’- AGAACTCTGGATGGGGAC-3’ |
| CDH17-F | 5’- TGGAGATGTAGTGAAAGG-3’ |
| CDH17-R | 5’- GAGACAACGAGTGTGAAG-3’ |
| KRT14-F | 5’- CCCAGTACTCCTCTGCAA-3’ |
| KRT14-R | 5’- CGGGATGAGACCACCTTC-3’ |
| CHST8-F | 5’- CCTTCATCTTGCTGTTTG-3’ |
| CHST8-R | 5’- CTGTTGCTACTCCGTCTG-3’ |
| OvoDA-F | 5’-TTCCTCTGCCTCGTCTTCG-3’ |
| OvoDA-R | 5’-AGCCCTTATTTCCACTTCC-3’ |
| OvoDB-F | 5’- TGCTCCTGCTCTTCTCCAT-3’ |
| OvoDB-R | 5’- CGACCACTCATCCTTTTTG-3’ |
| β-actin -F | 5’-TATGTGCAAGGCCGGTTTC-3’ |
| β-actin -R | 5’-TGTCTTTCTGGCCCATACCAA-3’ |

Note: F is the upstream primer, and R is the downstream primer.
