# Supplementary material for: Multiomic analysis revealed the regulatory role of the KRT14 gene in eggshell quality
Source: Front Genet. 2022 Sep 22;13:927670. doi: 10.3389/fgene.2022.927670 (PMC9536113; doi:10.3389/fgene.2022.927670)
Supplement: Supplementary file 3 [file Table2.DOCX]

Supplemental Table S2 Summary of sequencing results in chicken follicles at two stages

| sample name | Raw reads | Clean reads | Clean bases | Q20(%) | Q30(%) |
| --- | --- | --- | --- | --- | --- |
| Thick1 | 49,467,096 | 45,707,974 | 6.86G | 96.99 | 92.84 |
| Thick2 | 51,122,746 | 49,782,868 | 7.47G | 96.98 | 92.78 |
| Thick3 | 49,655,136 | 48,172,528 | 7.23G | 96.69 | 91.97 |
| Thin1 | 72,704,126 | 70,066,154 | 10.51G | 97.32 | 93.46 |
| Thin2 | 63,605,628 | 61,840,850 | 9.28G | 96.74 | 92.13 |
| Thin3 | 66,004,462 | 63,521,668 | 9.53G | 97.43 | 93.54 |
